# Comparison of transcutaneous electrical tibial nerve stimulation for the treatment of overactive bladder: a multi-arm randomized controlled trial with blinded assessment

**DOI:** 10.6061/clinics/2021/e3039

**Published:** 2021-08-05

**Authors:** Munick Linhares Pierre, Beatriz Friso, Raquel Aparecida Casarotto, Jorge Milhem Haddad, Edmund Chada Baracat, Elizabeth Alves Gonçalves Ferreira

**Affiliations:** I Departamento de Fisioterapia, Fonoaudiologia e Terapia Ocupacional, Faculdade de Medicina FMUSP, Universidade de Sao Paulo, Sao Paulo, SP, BR; II Departamento de Obstetricia e Ginecologia, Hospital das Clinicas HCFMUSP, Faculdade de Medicina, Universidade de Sao Paulo, Sao Paulo, SP, BR

**Keywords:** Overactive Bladder, Tibial Nerve, Transcutaneous Electrical Nerve Stimulation, Women’s Health, Physical Therapy Specialty

## Abstract

**OBJECTIVE:**

To compare the effectiveness of tibial nerve transcutaneous electrical nerve stimulation (TENS) for an overactive bladder, considering the sites of application and frequency of attendance.

**METHODS:**

This multi-arm randomized controlled trial enrolled 137 adult women (61.0±9.0 years) with overactive bladder from a university hospital. They underwent 12 sessions of 30-min TENS application and were assigned to five groups: one leg, once a week (n=26); one leg, twice a week (n=27); two legs, once a week (n=26); two legs, twice a week (n=28); and placebo (n=30). Symptoms of overactive bladder and its impact on quality of life were evaluated before and after 6 or 12 weeks of treatment using the Overactive Bladder Questionnaire-V8 and voiding diary. ClinicalTrials.gov: NCT01912885.

**RESULTS:**

The use of one leg, once a week TENS application reduced the frequency of urgency episodes compared with the placebo (1.0±1.6 *vs*. 1.4±1.9; *p*=0.046) and frequency of incontinence episodes compared with the placebo (0.7±1.4 *vs.*1.4±2.2; *p*<0.0001). The one-leg, twice a week protocol decreased the urinary frequency compared with the two legs, once a week protocol (8.2±3.5 *vs*. 9.0±5.1; *p*=0.026) and placebo (8.2±3.5 *vs*. 7.9±2.7; *p*=0.02). Nocturia improved using the two legs, once a week protocol (1.5±1.8) when compared with the one leg, twice a week protocol (1.9±2.0) and placebo (1.7±1.6) (*p*=0.005 and *p*=0.027, respectively). Nocturia also improved using the two legs, twice a week protocol when compared with the one leg, twice a week protocol (1.3±1.2 *vs.*1.9±2.0; *p*=0.011).

**CONCLUSION:**

One-leg stimulation improved the daily urinary frequency, urgency, and incontinence, and the two-leg stimulation once and twice weekly improved nocturia.

## INTRODUCTION

Transcutaneous electrical nerve stimulation (TENS) of the tibial nerve is a non-invasive, safe, low-cost, easy-to-apply method, with good adherence among patients, which helps to reduce symptoms of urgency, incontinence, daily urinary frequency, and nocturia (1-3). In the literature, authors describe good results with this therapy for treating urinary symptoms, in addition to enhanced quality of life and improved urodynamic measurements in patients with overactive bladder (4).

Many studies on transcutaneous electrical stimulation and urinary incontinence have sought to define optimal electrical parameters or protocols for treatment, although little is known about the dose of application or positions of the electrodes (5-8). A systematic review concluded that there is a need to determine the costs, most effective stimulation dosage, and the stimulation regimens for TENS in overactive bladder syndrome (9). There is no standardized dose for TENS applications, which varies from one to three times per week (10,11).

Because of the lack of consensus on the optimal dosage for assuring the effectiveness of TENS technique application in overactive bladder treatment, this study aimed to compare the effectiveness of transcutaneous electrical stimulation of the tibial nerve for overactive bladder, considering different sites and frequency of application.

## METHODS

This study was approved by the Ethics Committee of the Hospital das Clínicas, School of Medicine, São Paulo University (protocol no. 164.102) and was registered at www.ClinicalTrials.gov (Identifier: NCT01912885). This study did not receive any financial support. The study was conducted at the Urogynecology Physical Therapy Outpatient Clinic of the Hospital das Clínicas, School of Medicine, São Paulo University, between January and December 2015.

The participants were recruited from the urogynecology clinic. The inclusion criteria were: (1) female sex; (2) age >18 years; and (3) clinical diagnosis of overactive bladder at least six months before the study. Physicians who were not involved in the study diagnosed the subjects with an overactive bladder. The exclusion criteria were: (1) inability to understand the instructions given by the researcher; (2) pregnancy or desire to get pregnant soon; (3) urinary tract infection; (4) history of kidney stones; (5) stress urinary incontinence; (6) use of cardiac pacemakers; (7) drug treatment history for overactive bladder; (8) hormone replacement treatment performed in the previous six months; (9) peripheral neuropathy; (10) grade >2 cystocele; and (11) presence of epilepsy.

Patients were randomly assigned to five groups. The groups were named according to the site and frequency of application: one leg, once a week; one leg, twice a week; two legs, once a week; two legs, twice a week; and placebo. Randomization was performed using the website www.randomization.com by a researcher who was not involved in the intervention or evaluation. The random sequence of allocations was generated, with each allocation placed in a corresponding sequentially numbered opaque envelope. The envelopes were sealed and stored securely. The researcher responsible for the randomization informed only the physiotherapist who administered the interventions about each participant’s allocation.

The participants in all intervention groups received 30 min of TENS application. The electrodes were placed on either one leg (right) or both legs. The electrodes were placed along the tibial nerve course, one close to the medial malleolus and the other 10 cm above the first electrode. Patients were placed in a comfortable sitting position. The frequency of treatment was once or twice a week for 12 sessions, according to the group to which the patient was randomly assigned. The groups that received TENS once a week and placebo group ended the treatment within 12 weeks, while the twice a week groups completed their treatment within six weeks. We were interested in comparing the difference between once and twice a week instead of comparing the different numbers of sessions.

DUALPEX 961 (QUARK) was used to perform TENS. An electric current with a frequency of 10 Hz and pulse duration of 200 μs was programmed on the equipment. The intensity was adjusted according to the patient's tolerance; however, it was always kept below the motor threshold of the nerve (12).

The placebo group attended sessions once a week for 12 weeks. The electrodes were placed in one leg (right) for 30 min; however, the TENS remained switched off in the placebo group. The patients were not warned in advance that the equipment would be switched off. However, at the end of the study, the placebo group was invited to attend physical therapy treatment for an overactive bladder.

The assessments were performed at the beginning and end of the treatment. All assessments were performed by a physiotherapist who was blinded to the patients’ treatment groups. At the time of inclusion in the study, the patients were interviewed for anthropometric data, educational level, feeding behavior, physical activity level, and sexual activity. This is a routine evaluation performed at the outpatient clinic where the study was performed.

Symptoms of overactive bladder and the impact on quality of life were assessed with the Overactive Bladder Questionnaire in an abbreviated version (OAB-V8) (13), which has been previously validated in Portuguese language (14). The OAB-V8 included eight questions about the symptoms, with scores ranging from 0 to 40, in which higher scores meant worse symptoms (13,14). The question which the subject must answer refers to “how bothered one has been” by the urinary symptoms frequently observed in patients with overactive bladder, and the possible answers in this questionnaire are: “not at all,” “a little,” “somewhat,” “quite a bit,” “a great deal,” and “a very great deal.”

In addition, the symptoms of overactive bladder (urinary incontinence, urgency, and nocturia) and daily urinary frequency were recorded daily by the patients throughout the 12 sessions. The evaluator explained to the patient the difference between the symptoms and ensured that the patient had understood the information. Then, the patient was instructed to record the information daily according to the case, allowing the notes to be made using words, numbers, or another sign that symbolizes each symptom. The analysis was conducted considering the average days, dividing the number of occurrences of each event by the respective number of days.

All analyses were conducted using SAS System, version 9.0. The sample size calculation was based on the expected difference of three episodes in 24h (±3.25) among the groups on the voiding episode frequency at the end of the treatment (15). Considering 80% power and 95% confidence level, the sample size was estimated at 25 women per group. We added a possible sample loss of 20%, resulting in a sample size of 29 women per group and a total of 145 patients in the study.

The variables compared among the groups were daily urinary frequency, nocturia, urinary incontinence episodes, and urgency.

The chi-square test was used to assess whether there was an association between the sample description variables and treatments. The nonparametric Kruskal-Wallis test was used to compare treatments in terms of the difference between, before, and after the voiding diary variables. The analysis of variance (ANOVA) model for repeated measures was used to compare data from the specific voiding diary according to the treatment groups and sessions. Significant comparisons were evaluated using Tukey’s *post-hoc* test. Therefore, the comparison was performed group by group if the results from the ANOVA analysis were significant. To approximate the data to normality, a 1/log(variable+1) transformation was performed.

All statistical tests were performed per protocol based on a significance of 5%, that is, the null hypothesis was rejected when the *p*-value was <0.05.

## RESULTS

**Figure 1:**
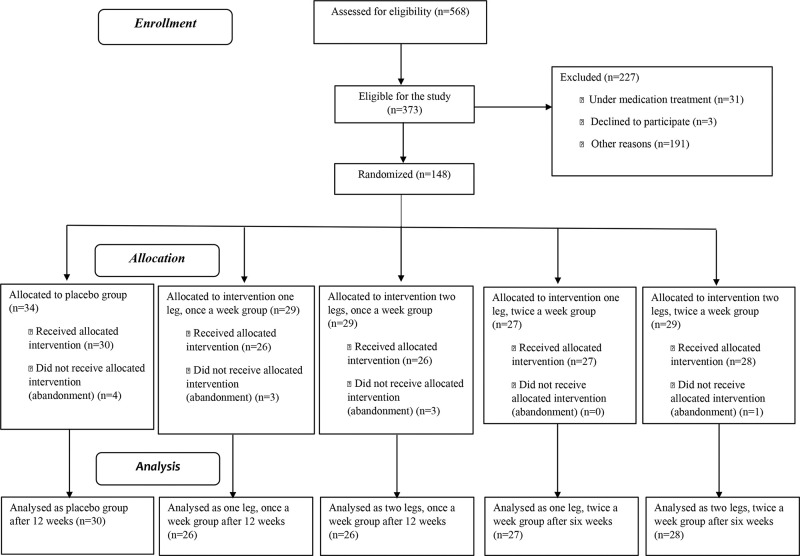
Flowchart of the patients enrolled in the study.

Five hundred and sixty-eight patients were initially assessed, and 148 were selected by randomization to participate in this study. Figure 1 presents details on the exclusion and analysis of patients. The descriptive analysis of the overall sample included 137 patients with a mean age of 61 years (standard deviation 9.0). The groups were initially similar in age (*p*=0.43), body mass index (*p*=0.12), number of pregnancies (*p*=0.62), and daily urinary frequency (*p*=0.53).

There were no differences in age, body mass index, and the number of pregnancies between the groups. We observed a high consumption of food that is irritant for detrusor muscle, such as caffeine, which was the most consumed item (74%) (Table 1).

There was no difference in the OAB-V8 scores between the groups after treatment (Table 2).

The daily urinary frequency showed improvement with the one leg, twice a week protocol compared with the two legs, once a week (8.2±3.5 *vs.* 9.0±5.1; *p*=0.026) protocol and placebo (8.2±3.5 *vs*. 7.9±2.7; *p*=0.020). In addition, nocturia improved using the two legs, once a week protocol (1.5±1.8) compared with the one leg, twice a week (1.9±2.0) protocol and placebo (1.7±1.6): *p*=0.005 and *p*=0.027, respectively. Nocturia also improved using the two legs, twice a week protocol, compared with the one leg, twice a week protocol (1.3±1.2 *vs*. 1.9±2.0; *p*=0.011). Furthermore, urgency improved with electrical stimulation using the one leg, once a week protocol, when compared with the placebo (1.0±1.6 *vs*. 1.4±1.9; *p*=0.046). Moreover, incontinence improved with the one-leg protocols. The one leg, once a week group was superior to the placebo (0.7±1.4 *vs.* 1.4±2.2; *p*<0.0001) and two legs, once a week groups (0.7±1.4 *vs.* 1.4±2.3; *p*<0.0001). The one leg, twice a week group was superior to the placebo (0.9±1.5 *vs.* 1.4±2.2; *p*=0.014) and two legs, once a week groups (0.9±1.5 *vs.* 1.4±2.3; *p*=0.037). The ANOVA results for these variables are presented in Table 2.

## DISCUSSION

In this study, we observed that patients who received TENS in one leg had improvement in the daily urinary frequency, urgency, and urinary incontinence. For nocturia, the best results were obtained with TENS applied in both legs. Application of TENS once a week was sufficient to achieve good results for most of the lower urinary tract symptoms. On the other hand, we did not observe any difference between the groups regarding the impact of overactive bladder on the quality of life measured by the questionnaire OAB-V8.

The results showed similarities in the clinical and demographic characteristics, confirming the initial homogeneity of the groups. In addition, the sample characteristics were consistent with the data from other studies (16,17).

Finazzi compared protocols of application of percutaneous electrical stimulation once or three times per week and concluded that the periodicity of stimulation did not affect the results of the treatment (18). In our study, we proposed to analyze not only the periodicity of electrical stimulation but also the number of legs in which the TENS was applied and our results suggest that different protocols may achieve better results depending on the outcome of interest. Wibisono and Rahardjo conducted a meta-analysis of 16 articles with percutaneous stimulation and observed that nine studies used protocols of once a week application, two studies used protocols of twice a week, three studies used protocols three times per week, one study used protocols one and three times per week, and only one study used a progressive protocol, reducing the frequency of application according to the proposed treatment (10). According to that meta-analysis, it appears that the increase in the number of days per week of TENS application is not a determinant factor for the success of the technique. Our results showed that weekly frequency of stimulation (once or twice a week) did not affect the treatment results and did not interfere with the improvement of symptoms, which is in accordance with the aforementioned studies; however, we demonstrated this result with transcutaneous electrical stimulation. Thus, the results may now be applied to this type of technique.

Electrical stimulation aims to improve the modulation of the detrusor nerve, with good results, safety, and no life-threatening adverse effects (19). Previous studies have tested tibial stimulation on the right leg and others on the left leg. However, to the best of our knowledge, this is the first trial to compare electrical stimulation on one leg with two legs (20). Our hypothesis was that electrical stimulation on two legs would enhance the known benefits for one leg; however, the results obtained in this study indicate that the stimulation of one leg is sufficient to improve the symptoms of an overactive bladder, except for nocturia, which presented the best result with the two legs protocol. We believe that more studies should be conducted to investigate the physiological mechanisms that justify this finding.

Transcutaneous electrostimulation is a widely used non-pharmacological treatment option for an overactive bladder. One of the objectives of this study was to evaluate the difference between having one or two sessions per week, for a total of 12 sessions. Reassessments were performed after 6 or 12 weeks of treatment. The difference in weeks in the total treatment time was also observed in other studies, depending on the research objective (11,19).

Chapple et al. concluded in their review that there is great difficulty in measuring overactive bladder results in clinical trials because of insufficient samples and lack of standardization in overactive bladder assessment (21).

Davila and Neimark reported that many factors can make it difficult to assess the quality of life in patients with urinary incontinence (22). In addition, a meta-analysis observed that different protocols using electrical stimulation reported improvement in the quality of life related to overactive bladder (20). Although designed for this type of population, the questionnaires that sought to assess the quality of life related to overactive bladder were unable to detect significant differences among the treated groups. We believe that this might have occurred because the voiding diary measures the number of events, that is, the number of times a person urinated during the day, number of times a person presented with incontinence episodes, and others, while the overactive bladder-V8 questionnaire evaluates the symptoms in a qualitative way. Also, of five groups, all received treatment and showed some improvement in the quality of life. In relation to the placebo group, an improvement of approximately 20% is expected, especially when a device such as TENS is used.

The results of this study can help physiotherapists to formulate an appropriate treatment regimen for overactive bladder in terms of the weekly frequency and number of electrodes used for TENS, since a wide range of physiotherapy techniques applied for several periods of time has been shown to be beneficial, but not for specific symptoms (20). The treatment, when conducted once a week, reduced symptoms in patients with overactive bladder and required half of the workload for both patients and therapists. Transcutaneous electrical stimulation of the tibial nerve once per week can be considered a good treatment option.

This study had some limitations. The number of follow-up losses was foreseen in the initial protocol. Unfortunately, most women were not contacted promptly for follow-up and we preferred not to continue follow-up because of the risk of bias. However, in this manner, we cannot present long-term follow-up data. The use of a three-day voiding diary was provided in the protocol of this study. However, almost all patients were unable to complete the diary because of a lack of understanding, even after the guidance provided by the evaluator. This is possibly because of the low level of education in the population included in this study. Finally, since we have five groups, there is the risk of multiple comparisons in the statistical analysis. Also, for the sample size calculation, we used a two-arm study, which may reduce the power of the study.

The design of this study can be considered a strength because it is a multi-arm randomized controlled trial with blinded assessment. The conclusion of this study points to the fact that TENS must be performed twice a week in specific situations, which impacts the cost of treatment.

The application of TENS in patients with an overactive bladder should be adapted according to the specificity of the symptoms reported by the patient. More studies are needed, especially concerning the long-term effects of the technique, although the present study suggests that TENS therapy should be performed according to each patient's symptoms.

## CONCLUSION

The one leg, once a week TENS protocol presented better results concerning the treatment of symptoms of daily urinary frequency, urgency, and incontinence. However, for nocturia symptoms, the two-leg protocol was more effective. This study highlights the importance of investigating the physiotherapeutic treatment for each specific symptom of an overactive bladder.

## AUTHOR CONTRIBUTIONS

Pierre ML was responsible for the study design, data collection and analysis, and manuscript writing. Friso B was responsible for the study design, data collection, and manuscript review. Casarotto RA, Haddad JM, Baracat EC and Ferreira EAG were responsible for the study design, data analysis, and manuscript review.

## Figures and Tables

**Table 1:** Baseline characteristics of patients with overactive bladder included in the study (n=137) according to the randomized groups.

Characteristics	One leg, once a week group (n=26)	Two legs, once a week group (n=26)	One leg, twice a week group (n=27)	Two legs, twice a week group (n=28)	Placebo group (n=30)
Age, years	58±9	62±9	62±7	62±10	60±11
Body mass index, kg/m^2^	26±6	30±6	29±7	29±4	29±5
Number of pregnancies	3.2±2.2	3.8±2.8	3.1±1.7	3.8±1.8	3.8±2.7
Post-menopause, n (%)	22 (85)	25 (96)	23 (85)	26 (93)	29 (97)
Sexually active, n (%)	12 (46)	8 (31)	8 (30)	11 (39)	12 (40)
Sedentary, n (%)	17 (65)	22 (85)	17 (63)	18 (64)	21 (70)
Married or with partner, n (%)	11 (42)	12 (46)	14 (52)	15 (54)	12 (40)
Educational level, n (%)					
<4 years	2 (8)	2 (8)	2 (7)	0	2 (7)
4-8 years	16 (62)	15 (58)	19 (70)	13 (46)	15 (50)
9-11 years	5 (19)	6 (23)	5 (19)	10 (36)	12 (40)
≥12 years	3 (12)	3 (12)	1 (4)	5 (18)	1 (3)
Frequency of initial urinary symptoms					
Daily urinary frequency	11.0±6.3	10.5±8.0	9.6±4.0	9.1±3.0	8.9±3.9
Nocturia	1.9±1.3	1.8±1.6	2.8±2.2	2.0±1.3	2.6±1.9
Urinary urgency	3.2±2.1	3.3±3.0	2.8±2.4	3.8±2.4	3.0±2.4
Incontinence	1.8±1.5	2.8±2.5	2.0±1.6	2.2±1.8	2.6±2.2
Initial overactive bladder-V8	24.4 (20.2-28.7)	26.5 (20.5-31.0)	29.0 (16.5-32.0)	27.0 (24.5-31.0)	27.0 (22.0-31.7)

Overactive bladder-V8: Overactive Bladder Questionnaire in an abbreviated version. Initial urinary symptoms were collected from a specific voiding diary at the beginning of the treatment. Overactive bladder-V8 was collected using a questionnaire at the beginning of the treatment. Data are presented as means±standard deviations and medians (1^st^-3^rd^ quartiles) unless otherwise indicated.

**Table 2:** Descriptive analysis of the score using the Overactive Bladder Questionnaire in an abbreviated version and the urinary symptoms obtained from specific voiding diary, both at the end of treatment^#†^ (n=138).

Variables	One leg, once a week group^#^ (n=26)	Two legs, once a week group^#^ (n=26)	One leg, twice a week group^†^ (n=27)	Two legs, twice a week group^†^ (n=28)	Placebo group^#^ (n=30)	p*
OAB-V8 score	7.5 (3.0≥14.5)	11.0 (2.0-21.2)	5.0 (0-19.0)	11.0 (1.0-21.5)	12.5 (3.0-25.0)	0.847
Specific voiding diary						
Daily urinary frequency	8.4±3.9	9.0±5.1	8.2±3.5^a^	8.2±2.6	7.9±2.7^b^	0.018
Nocturia	1.6±1.3	1.5±1.8^c,d^	1.9±2.0	1.3±1.2^e^	1.7±1.6	0.000
Urinary urgency	1.0±1.6^f^	1.3±2.2	1.2±1.8	1.2±1.9	1.4±1.9	0.081
Incontinence	0.7±1.4^g,h^	1.4±2.3	0.9±1.5^i,j^	0.9±1.4	1.4±2.2	<0.001

Overactive bladder-V8: Overactive Bladder Questionnaire in an abbreviated version. Data are presented as medians (1^st^-3^rd^ quartiles) for the overactive bladder-V8 and as means±standard deviations for the specific voiding diary features.^ #^The treatment ended after 12 weeks. ^†^Treatment ended after six weeks. *p-values for the comparison among groups using Kruskal Wallis test for the overactive bladder-V8 and ANOVA for the specific voiding diary features. The *p*-values for the *post-hoc* Tukey tests for the specific voiding diary features are as follows: ^a^One leg, twice a week *vs.* two legs, once a week, *p*=0.026. ^b^One leg, twice a week *vs*. placebo group, *p*=0.020. ^c^Two legs, once a week *vs*. one leg, twice a week, *p*= 0.005. ^d^Two legs, once a week *vs.* placebo group, *p*=0.027. ^e^One leg, twice a week *vs.* two legs, twice a week, *p*=0.011. ^f^One leg, once a week *vs.* placebo group, p=0.046. ^g^One leg, once a week *vs.* two legs, once a week, *p*<0.0001. ^h^One leg, once a week *vs.* placebo group, *p*<0.0001 ^i^One leg, twice a week *vs.* two legs, once a week, *p*=0.037. ^j^One leg twice a week *vs*. placebo group, *p*=0.014.
